# Patidegib in Dermatology: A Current Review

**DOI:** 10.3390/ijms221910725

**Published:** 2021-10-03

**Authors:** Terenzio Cosio, Monia Di Prete, Cosimo Di Raimondo, Virginia Garofalo, Flavia Lozzi, Caterina Lanna, Emi Dika, Augusto Orlandi, Maria Cristina Rapanotti, Luca Bianchi, Elena Campione

**Affiliations:** 1 Dermatologic Unit, Department of Systems Medicine, University of Rome Tor Vergata, Via Montpellier, 1 00133 Rome, Italy; terenziocosio@gmail.com (T.C.); cosimodiraimondo@gmail.com (C.D.R.); virginia.garofalo27@gmail.com (V.G.); flavia.lozzi@hotmail.com (F.L.); caterinalanna.cl@gmail.com (C.L.); luca.bianchi@uniroma2.it (L.B.); 2 Anatomic Pathology, University of Rome Tor Vergata, Via Montpellier, 1 00133 Rome, Italy; diprete.monia@gmail.com (M.D.P.); orlandi@uniroma2.it (A.O.); 3 Anatomic Pathology, Santa Maria di Ca’ Foncello Hospital, 31100 Treviso, Italy; 4 Division of Dermatology, Department of Experimental, Diagnostic and Specialty Medicine (DIMES), University of Bologna, 40138 Bologna, Italy; emi.dika3@unibo.it; 5 Dermatology, IRCCS Policlinico di Sant’Orsola, Via Massarenti 9, 40138 Bologna, Italy; 6 Laboratory Medicine, University of Rome Tor Vergata, Via Montpellier, 1 00133 Rome, Italy; cristinarapanotti@yahoo.it

**Keywords:** basal cell carcinoma, hedgehog signaling inhibitors, IPI-926, patidegib, targeted cancer therapy

## Abstract

Background: Basal cell carcinoma is one of the most common types of non-melanoma skin cancers, which can be locally destructive despite low-rate metastasis. Surgery is the treatment of choice, but it lacks of efficacy on advanced cases. Hedgehog pathway inhibitors are a class of drugs providing a new therapeutic option for patients affected by advanced disease. Besides systemic therapy, such as vismodegib and sonidegib, also topical inhibitors have been developed. Patidegib is able to decrease tumor burden, reducing the adverse effects induced by systemic targeted therapies. Methods: We performed comprehensive research to summarize the use of patidegib in advanced and recurrent aggressive basal cell carcinomas. Only English language human studies were included in the search. Results: Seven trials reported the application of patidegib. Both topical and systemic patidegib demonstrated safety, tolerability, and efficacy in naïve patients with stage II and III basal cell carcinomas, while stage IV disease and not-naïve patients did not show any benefit. Conclusion: Unlike systemic Hedgehog pathway inhibitors, patidegib 2% gel is not associated with systemic adverse effects and allows a better patient management. Considering the multidisciplinary management of neoplasia, in the era of precision medicine, it is mandatory to confide in pharmacogenomics to obtain personalized combined or sequential therapies.

## 1. Introduction

**Figure 1:**
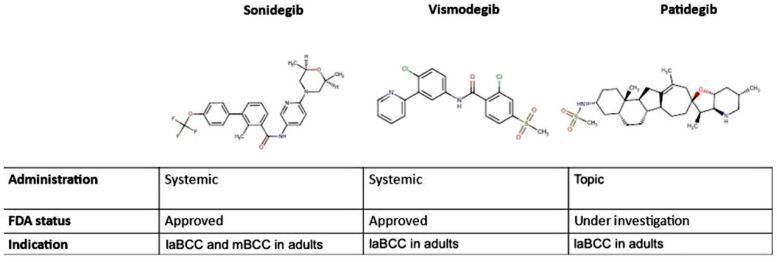
Hedgehog pathway inhibitors comparison. Molecular structures, administration, FDA status and indication of sonidegib, vismodegib, and patidegib. laBCC, local advanced Basal Cell Carcinoma; BCC basal cell carcinoma.

Basal cell carcinoma (BCC) is the most common epidermal malignancy, and its incidence is rising. BCCs have a low mortality rate, but can cause significant morbidity, primarily through local destruction. The pathogenesis is linked to the interplay between environment and patient’s phenotypes [1]. There are multiple therapeutic modalities, and the specific choice in each case requires the knowledge of complications, cosmetic outcomes, and recurrence rates [2]. Advanced BCC (aBCC) is more aggressive than conventional BCC and topical therapies, that are routinely used, are not effective [3,4,5,6,7]. Hedgehog pathway (Hp) signalling is involved in regulating proliferation, stem cell population, differentiation, tissue polarity, and carcinogenesis, particularly in the arising of aBCC [8,9,10] In mammals, Hp signaling molecules include three ligands (Sonic, Indian, and Desert hedgehog), two receptors named Protein Patched Homolog (PTCH1, and PTCH2), a crucial signal transducer smoothened (SMO) and three zinc-finger proteins, named glioma-associated oncogene (Gli1, Gli2, and Gli3), which act as transcription factors [11]. Until Hp ligands are not present, the SMO function is blocked by PTCH1 and PTCH2. Inactivating mutations of PTCH1 result in constitutive Hp activity through uncontrolled SMO signaling. These eventslead to the nuclear translocation of Gli1 and Gli2, with consequent transcription of Hp responsive genes [12]. Hp is physiologically responsible of normal embryo development, butin patients with Gorlin syndrome, PTCH mutations cause unrestrained signaling through SMO, with high rate of tumor, especially BCCs [13]. The discovery of cyclopamine, the first natural Hp inhibitor, allowed to study widely the pathway and led to identify SMO as an effective target to down-regulate it [14]. Because of the teratogenicity of cyclopamine, the need to find other Hp inhibitors with a better safety profile became soon clear.. This research headed to the development of vismodegib (GDC-0449) and sonidegib (NVP-LDE225) [15,16], synthetic derivatives of cyclopamine. Vismodegib has been approved for aBCCs and recurrent BCCs (rBCCs) since 2012, while sonidegib has been employed for locally advanced BCCs (laBCCs) since 2015 [17,18]. Targeting Hp, vismodegib triggers tumor regression inpatients with these genetic mutations [19]. Nowadays, a new semisynthetic Hp inhibitor, patidegib, also known as saridegib or IPI-926, an experimental drug undergoing clinical trials for the treatment of aBCC, is showing promising results (Figure 1) [20]. Patidegib exhibits its pharmacological effect by inhibiting SMO [20], and received approval as Orphan Drug Designation and Breakthrough Therapy Designation as topical gel in Gorlin syndrome both by FDA and EMA’s Committee for Orphan Medicinal Products in the EU in 2018 [21,22]. The aim of this review is to highlight the efficacy and tolerability of patidegib in aBCCs and rBCCs.

## 2. Methods and Study Design

### 2.1. Search Strategy

We performed a comprehensive search in the following databases from 2009 to 2020: Cochrane Central Register of Controlled Trials; MEDLINE; Embase; US National Institutes of Health Ongoing Trials Register; NIHR Clinical Research Network Portfolio Database; and the World Health Organization International Clinical Trials Registry Platform. We studied reference lists and published systematic review articles. We used the following keywords in combination: “patidegib”, “saridegib”, “IPI-926”, “Gorlin syndrome”, “basal cell carcinoma”, “dermatology”. The search was restricted to studies on humans and only English language articles were included.

### 2.2. Inclusion Criteria

To investigate the use of systemic and topical patidegib in dermatological disorders, if a study included patidegib with other drugs, only the patidegib frame was analyzed. All human studies were included with no restrictions on age, sex, ethnicity, or type of study. Case reports and case series were included if they described the use of patidegib in diseases not present in reviews or trials.

### 2.3. Exclusion Criteria

The target intervention excluded the analyses of other pathologies not belonging to the dermatological field, animal studies, and non-English language articles.

## 3. Results

We identified forty-six articles and trials regarding the use of patidegib. Thirty-six were excluded due to the exclusion criteria, while three were excluded after evaluating the trials. A total of seven studies has been included in this comprehensive review (Figure S1). Patidegib was granted FDA Orphan Drug and Breakthrough Therapy Designation for the treatment of Gorlin Syndrome and by EMA in 2018 [22].

### 3.1. Phase I Trials

Jimeno et al. reported the first-in-human phase I study to determine the dose-limiting toxicities, characterize the pharmacokinetic profile, and document the anti-tumor activity of patidegib in solid tumors [23]. A 20 mg single-dose oral patidegib was administered, to evaluate the drug pharmacokinetic profile, 7 days before beginning a 28-day treatment cycle of daily oral patidegib, in an outpatient setting. They presented 39 subjects affected by aBCCs, 5 of whom were affected by Gorlin’s syndrome. Ten patients were stage II, ten were stage III, and nineteen were stage IV (Table S1). 28 patients were evaluated (as they received more than one dose of treatment and had a post-baseline assessment) and were vismodegib-naïve. Patidegib showed substantial anti-tumor activity in these patients. Two patients achieved complete response, while 6 had partial response. All these patients had laBCC. In this study were reported also two patients who received patidegib for 50 and 18 weeks, respectively, after progression during vismodegib treatment. Interestingly, they did not experience any objective response. Thus, patidegib showed substantial anti-tumor activity in patients with vismodegib-naïve BCCs, confirming the specificity of the drug for the same molecular target. However, disease progression ultimately developed, in a time similar to other tumors known to develop secondary mutations with other targeted agents [24,25]. In summary, patidegib showed single-agent activity in patients with BCCs and a manageable toxicity profile that was consistent with its class.

Zhu et al. reported a case of Gorlin syndrome in a patient with biopsy-proven metastatic BCC to the lung that was refractory to two different SMO inhibitors despite responses in his cutaneous lesions [26]. All his cutaneous BCCs responded to patidegib. However, his metastatic BCCs remained stable in size on CT scans, indicating a refractory disease. Because of his comorbidities and lack of treatment options, the patient was enrolled in a clinical study (NCT01160250) using vismodegib, after 1-month washout from patidegib. This case demonstrates that the treatment responses of distant metastatic BCCs may not reflect the treatment responses of cutaneous BCCs in patients with Gorlin syndrome. Although this patient’s cutaneous BCCs responded to SMO inhibitors, his metastatic disease progressed and developed new nodules. Moreover, these results highlight a possible common resistance mechanism involving both vismodegib and patidegib, excluding a potential proficient sequential therapy.

### 3.2. Phase II Trials

#### 3.2.1. Completed 

A multicenter, double-blind, randomized, vehicle-controlled clinical trial (NCT02762084) evaluated the efficacy and safety of patidegib 2% and 4% gel compared to vehicle gel in patients affected by Gorlin syndrome with stage I BCCs [27]. Participants were randomized to receive patidegib 2% gel, patidegib 4% gel, or the vehicle gel twice daily for 26 weeks of treatment. After 26 weeks, patidegib 2% gel showed a reduction of 51.29% in the number of tumors from baseline, while patidegib 4% gel shown a reduction of 26.63%, demonstrating a better clinical effect at 2% vs. 4% concentration. A double-blind, dose escalating, randomized, vehicle-controlled clinical trial (NCT02828111) was designed to compare the efficacy and safety of patidegib 2% and 4% gel applied once or twice daily for 12 weeks compared to that of vehicle in patients with BCC [28]. The data showed that only two of the six patients who received patidegib 4% gel twice daily had local adverse effects (AEs). Molecular efficacy was evaluated as percentage change from baseline in the Hp signaling target gene, GLI1, messenger RNA (mRNA) levels at the end of the treatment. The best response was obtained by patidegib 2% gel once daily, with a reduction of −56.3% as change in the GLI1mRNA levels from baseline (Table S2).

The results of both trials demonstrated that patidegib 2% gel has a higher clinical and molecular efficacy on BCC, with less AEs, than the 4% gel (Table 1).

#### 3.2.2. Uncompleted

A multicenter, randomized, double-blind, stratified, vehicle-controlled study (NCT04155190) on efficacy and safety of patidegib 2% gel topically applied twice daily on the face of adult participants with non-Gorlin syndrome associated high-frequency BCCs is currently in the recruiting phase (Table S3). Participants are going to be randomized (1:1) to receive patidegib 2% gel or vehicle gel for 9 months. Endpoints are going to be assessed by imaging and tracking of BCCs consistently throughout the study in order to identify surgically eligible BCCs [29].

### 3.3. Phase III Trials 

To data, two ongoing phase III trials (Table S3) in patients with Gorlin syndrome are evaluating patidegib 2% gel (NCT04155190) and the reduction of disease burden of persistently developing BCCs (NCT04308395) [29,30]. Moreover, a phase III trial is recruiting participants to evaluate patidegib 2% gel for the reduction of disease burden of persistently developing BCCs in patients with non-Gorlin syndrome high frequency BCCs (NCT03703310) [31].

In all these trials, the rational use of topical patidegib 2% gel is based on previous results demonstrating superior clinical and molecular efficacy of 2% vs. 4% concentration [27,28].

## 4. Discussion

### 4.1. Histopathological Considerations

For most BCCs, complete surgical excision, associated with histopathological examination, is both diagnostic and therapeutic [1]. On the other hand, a small percentage of lesions is at high risk of recurrence, such as laBCC, which is defined as a tumor ≥ 2 cm, without lymph nodes involvement nor distant metastasis. In these cases, Mohs’ micrographic surgery is the preferred technique, as it allows to evaluate margins status during the procedure [8]. Nonsurgical topical therapies are generally not used due to disease or patient-related factors. Among the former factors, there are tumor size and location (i.e., the face, where is sometimes difficult to obtain adequate free margins), number of lesions, and histotype (i.e., high-risk recurrence variants). The latter factors include age, general performance status, and comorbidities [8]. Despite the heterogeneity in their clinical presentation, the histopathological appearance of BCC is common to all histotypes. Basaloid cells, presenting hyperchromatic nuclei, without evident nucleoli, and scant cytoplasm, are organized in nests and/or islands, with peripheral palisading. The aggregates are surrounded by a loose fibrous stroma, which often presents myxoid changes, and are responsible for lack of cohesion between the tumor nests and its stroma, with the consequent characteristic retraction artifact after formalin fixation [8]. The different histopathological BCC variants, as defined by the latest WHO Classification of the Skin Tumors, combined with clinical presentations and response to treatment, allow to stratify the risk of BCC recurrence, as a formal staging system for this disease is not available yet. Low-risk variants are superficial, pigmented, nodular, with adnexal differentiation, and fibroepithelioma of Pinkus. High-risk histotypes include infiltrating, micronodular, morphoeic/sclerosing, basosquamous carcinoma, and sarcomatoid differentiation. From a histopathological point-of-view, the differential diagnoses include trichoepithelioma and squamous cell carcinoma (SCC) with basaloid features. Immunohistochemical stains have an important role in this setting. BCC shows a stronger and diffused Bcl-2 and CD10 expression more frequently than trichoepithelioma, while the latter is positive for CK20 and podoplanin (D2-40) in the peripheral tumor nests [8]. Finally, BerEP4 (EpCAM) and epithelial membrane antigen may be helpful in the distinction between BCC and basaloid SCC, as the former is positive for BerEP4 and does not express the epithelial membrane antigen, and vice versa.

### 4.2. Inefficacy of Sequential Hp Inhibitors Therapy

Tumor resistance mechanisms have been already largely documented. SMO mutations secondary to anti-SMO therapy have been reported to occur and cause treatment resistance [32]. SMO mutations are responsible for resistance both to vismodegib and sonidegib in up to 50% of aBCC cases [33,34]. Together with SMO G497W mutation, which causes primary resistance to vismodegib [33], competition binding assays detected other mutations triggering significant modification in the binding affinity between SMO protein and Hp inhibitors [35]. The most common is the D473A, which determines a significant decrease in binding affinity for both vismodegib and sonidegib, while E518A leads to a drop in the affinity for vismodegib and a slender increase for sonidegib [35,36]. Furthermore, through computational docking studies of vismodegib onto SMO, it has been demonstrated that the mutations W281, V321, I408, and C469 are located near the drug-binding pocket, decreasing the drug affinity for its molecular target [37]. Lastly, modifications in GLI1 gene copy number or mutations of suppressor of fusion (SUFU), determining its loss-of-function, have also been involved in vismodegib resistance [36]. Jimeno et al. highlighted the efficacy of patidegib in naïve patients, while vismodegib pre-treated patients have no clinical improvement of BCCs [23]. Danial et al. evaluated aBCCs response to sonidegib in patients previously treated with vismodegib. Their conclusion reported that patients with aBCCs resistant to vismodegib similarly demonstrated treatment resistance to sonidegib. Moreover, patients who developed resistance to a SMO inhibitor may continue to experience tumor progression also in response to other SMO inhibitors [38]. These data highlight a cross-resistance among the same class of drugs, which included also patidegib. The same results have been reported also by Zhu et al., who described a patient with mBCC treated with patidegib and then with vismodegib, who experienced disease progression [26]. Thus, sequential therapies with Hp inhibitors is not indicated, while more therapeutic options can be evaluated for immunotherapy and targeted sequential therapies (i.e., targeted therapy and sequential immunotherapy in metastatic melanoma).

### 4.3. Patients with Comorbidities

Frailty and comorbidities are age-related clinical manifestations representing diminished functional reserve and accumulation of pathological processes, respectively, leading to an impaired quality of life. They often overlap in elderly patients with the need y of having multiple medications at the same time [39]. In this scenario, vismodegib and sonidegib may have a limited role as their systemic AEs could be amplified by the interaction with other drugs. First of all, vismodegib undergoes hepatic metabolism by oxidation, glucuronidation, and ring cleavage. In elderly patients, liver dysfunction or extra-hepatic pathologies could influence the pharmacodynamics of systemic medications [40]. Moreover, vismodegib-associated AEs may be detrimental to long-lasting treatment. Each patient in the ERIVANCE trial experienced at least one AE, which in most cases was of mild to moderate in entity [41]. Only 13 individuals (12%) had an AE that ultimately led to the discontinuation of vismodegib. The most common vismodegib-related AEs consist in muscle spasm (72%), alopecia (64%), dysgeusia (55%), weight loss (45%), and fatigue (40%). Other less frequent AEs include nausea, diarrhea, constipation, decreased appetite, ageusia, and arthralgias. Rarely, patients may experience electrolyte disturbances, including hypokalemia or hyponatremia. Additionally, there may be elevated creatine phosphokinase levels and azotemia [41]. A retrospective cohort study made by the University of California in San Francisco evaluated the risk of developing SCC following the use of vismodegib. They determined the absence of the risk compared to standard surgical treatment for BCC. However, further studies are needed to examine this topic [42]. On the other hand, sonidegib is also metabolized in the liver, via the cytochrome P450 (CYP) enzyme, CYP3A4. The main circulating compounds are unchanged sonidegib (36%) and its metabolites (45%) [43]. Nonetheless, based on pharmacokinetic observations, no dose adjustment is needed for mild and moderate renal or mild hepatic impairment, but patients have to be monitored closely for adverse events [44]. As reported for vismodegib, also sonidegib could cause muscle spasms, alopecia, dysgeusia, fatigue, nausea, diarrhea, decreased weight, decreased appetite, and myalgia [44]. In this context, patidegib topical formulation could represent a first choice of treatment in fragile patients with comorbidities, to avoid systemic AEs. Moreover, topical self-application allows a better home-management of the medication, thus a higher treatment compliance. Topically applied patidegib demonstrated no systemic absorption and, in a phase III clinical trial, the number of patidegib causally-related AEs (including both severe and non-severe events) was low, 2/23 patients, compared to zero in the vehicle arm of the study. In particular, AEs were reported in the patidegib 4% gel arm, applied topically twice daily for 12 weeks, while all the other arms of the study (consisting in patidegib 2% and 4% gel, applied once daily for 12 weeks and patidegib 2% gel applied twice daily for 12 weeks) reported no AEs [27]. These results support the safety and effectiveness of patidegib 2% gel 2% topically applied once daily for 12 weeks.

### 4.4. Perspectives

Recent and upcoming investigations on targeting Hp signaling in tumors should concentrate on strategies addressing and exceeding resistance mechanisms. The design and development of new generation therapeutics should take into account both downstream genetic variants, such as GLI gain-of-function and SUFU loss-of-function mutations, and acquired SMO mutations [45,46]. In a recent proof of concept, we speculated on the association of, itraconazole, arsenic trioxide, all-trans-retinoic acid and nicotinamide combined with conventionalHp inhibitors to carry out the therapeutic response, prevent tumor resistance and diminish the dosage of each drug and associated AE [9]. To data, no actual combined or sequential therapies with patidegib have been described in available trials. Considering the multidisciplinary management of neoplasia, in the era of precision medicine, it is mandatory to confide in pharmacogenomics to obtain personalized combined or sequential therapies.

## 5. Conclusions

Unlike systemic Hp inhibitors, topical patidegib avoids systemic AEs and allows a better patient management, especially for fragile patients. To data, both topical and systemic patidegib have demonstrated safety, tolerability, and efficacy in naïve patients with stage I, II, and III cutaneous BCCs, both correlated and not correlated with Gorlin syndrome, while stage IV disease and not-naïve patients did not show any benefit.

## Figures and Tables

**Table 1:** The table shows the results of the two phase II trials evaluating patidegib use in basal cell carcinomas. Clinical and molecular efficacy are compared. Clinical efficacy is calculated as percentage change in the greatest diameter of treatment-targeted surgically eligible basal cell carcinomas (SEBs) from baseline to the end of treatment (which is indicated for each trial and drug posology). SEBs were defined as clinically diagnosed BCC of 5 to 20 mm in diameter on the face, excluding the nose and periorbital skin, and of 9 to 20 mm on sites other than the face. Molecular efficacy is evaluated as percentage change in Hedgehog pathway signaling target gene Glioma-associated oncogene homolog 1 (GLI1) messenger RNA levels. Abbreviations: Investigator Static Global Tumor Assessment.

	Drug Formulation	Timing (Daily; Weeks)	Clinical Outcome	Molecular Outcome	ISGTA Scale
**NCT 02828111**	Patidegib 2% gel	Once; 12	−56.15% (48.14)	−56.3% (99.59)	42.9
		Twice; 12	−17.01% (36.87)	−42.51% (55.64)	20.0
	Patidegib 4% gel	Once; 12	−8.73% (46.6)	−3.24% (69.03)	0
		Twice; 12	−18.41% (60.59)	−28.85% (46.23)	16.7
**NCT 02762084**	Patidegib 2% gel	Twice; 26	−51.29% (41.78)	−53.83% (27.2)	33.3
		Twice; 14	N/A	N/A	33.3
	Patidegib 4% gel	Twice; 26	−26.63% (41.27)	−20.69% (34.73)	30.0
		Twice; 14	N/A	N/A	13.3

## Data Availability

Data are available on reasonable request.

## Supplementary Materials

The following are available online at https://www.mdpi.com/article/10.3390/ijms221910725/s1.
