# Supplementary material for: Patidegib in Dermatology: A Current Review
Source: Int J Mol Sci. 2021 Oct 3;22(19):10725. doi: 10.3390/ijms221910725 (PMC8509734; doi:10.3390/ijms221910725)
Supplement: Supplementary file 1 [file ijms-22-10725-s001.zip › ijms-1365130-supplementary.pdf]

Supplementary Materials

Table S1. Case report and series not present on ClinicalTrials.gov reporting phase I trials results (Abbreviations: BCC; basal cell carcinoma).

**Publication title (author)**	**Drug evaluated**	**Sample size and disease staging**	**Study results**
Phase I Study of the Hedgehog Pathway Inhibitor IPI-926 in Adult Patients with Solid Tumors (Jimeno et al.)	Patidegib 20 mg; 28-day cycles	39; stages II, III and IV naïve and not-naïve BCCs	2 patients achieved complete response; 6 partial response; 2 not-naïve did non experienced any response
Patient with Gorlin syndrome and metastatic basal cell carcinoma refractory to smoothened inhibitors (Zhu et al.)	Patidegib 130mg/die for 16 months	1; stage IV not-naïve BCC	Cutaneous BCC responded, while metastatic disease progressed

Table S2. Completed trials reporting results for patidegib use in basal cell carcinomas.

**Official Title on ClinicalTrials.gov or publication title**	**Phase**	**Sample size**	**Study results**
Double-Blind, Randomized, Vehicle-Controlled Proof of Concept Clinical Trial of Patidegib Gel 2%, 4%, and Vehicle to Decrease the Number of Surgically Eligible Basal Cell Carcinomas in Gorlin Syndrome Patients (NCT 02762084)	II	17 participants; Randomized Parallel Assignment	Twice daily; 26 weeks:<br>- patidegib 2% gel 51.29% clinical and 53.83% molecular reduction;<br>- patidegib 4% gel 26.63% clinical and 20.69% molecular reduction
Double-Blind, Dose Escalating, Randomized, Vehicle-Controlled Proof of Concept Clinical Trial of Patidegib Gel 2%, 4%, and Vehicle Applied Once or Twice Daily to Decrease the	II	36 participants; Randomized Parallel Assignment	Patidegib 2% gel once daily for 12 weeks: 56.15% clinical and 56.3% molecular reduction;<br>Patidegib 4% gel twice daily for 12 weeks: 18.41% clinical and 28.85% molecular

GLI1 Biomarker in Sporadic Nodular Basal Cell Carcinomas (NCT 02828111)	reduction

Table S3. Ongoing trials regarding patidegib use in basal cell carcinomas. Study results are still not available (Abbreviations: BCC; basal cell carcinoma).

Official Title on ClinicalTrials.gov or publication title	Phase	Drug evaluated	Sample size
A Multicenter, Randomized, Double Blind, Vehicle-controlled, Phase 2 Efficacy and Safety Study of Patidegib Topical Gel, 2%, for the Reduction of Disease Burden of Persistently Developing Basal Cell Carcinomas in Patients With Non-Gorlin High Frequency BCC (NCT 04155190; recruiting)	II	Patidegib 2% gel	45 patients; Randomized Parallel Assignment
A Phase 3, Multicenter, Open-Label Extension Study of Patidegib Topical Gel, 2% in Subjects With Gorlin Syndrome (Basal Cell Nevus Syndrome) (NCT 04308395; enrolling by invitation)	III	Patidegib 2% gel	191 patients; Single Group Assignment
A Multicenter, Randomized, Double-blind, Vehicle-controlled, Phase 3 Efficacy and Safety Study of Patidegib Topical Gel, 2%, for the Reduction of Disease Burden of Persistently	III	Patidegib 2% gel	174 patients; Randomized Parallel

Developing Basal Cell Carcinomas (BCCs) in Subjects With Basal Cell Nevus Syndrome (NCT 03703310; active, not recruiting)	Assignmen t

Figure legends.

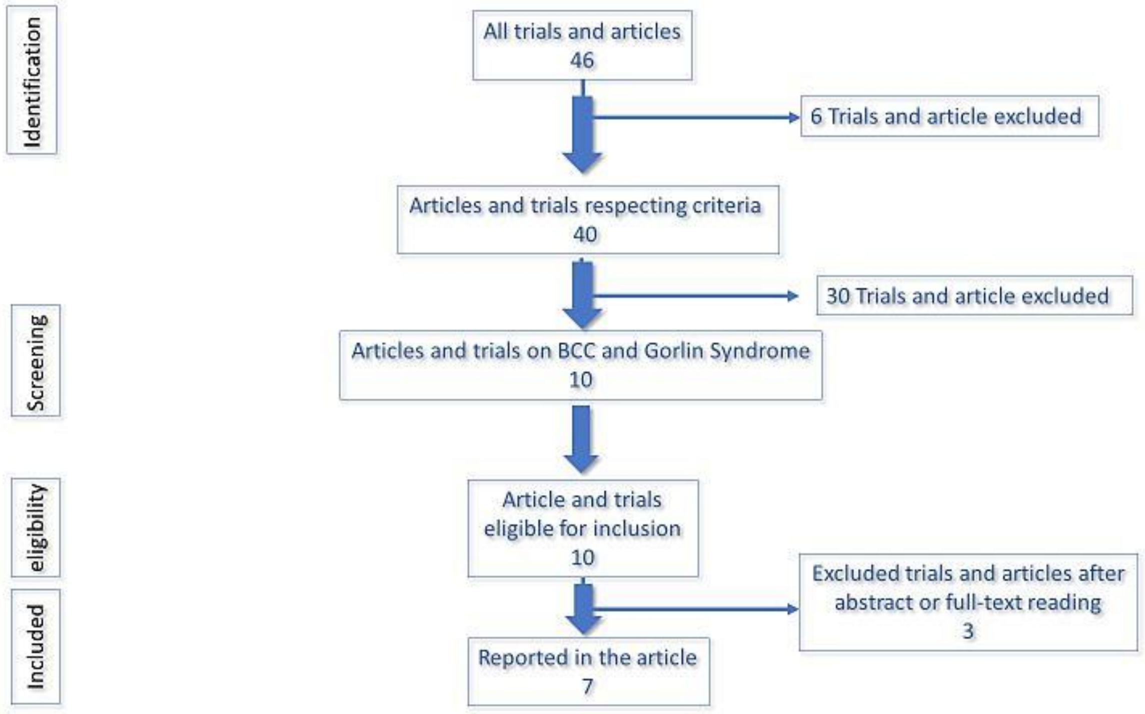

Figure S1. Flowchart summarizing the research strategy to select clinical trials and articles about patidegib use in basal cell carcinomas (Abbreviations: BCC; basal cell carcinoma).
